# Synthesis and Characterization of LaNi_0.5_Ti_0.5_O_3_ and La_2_NiTiO_6_ Double Perovskite Nanoparticles

**DOI:** 10.3390/ma15072411

**Published:** 2022-03-25

**Authors:** José Córdova-Calderón, Pablo V. Tuza, Mariana M. V. M. Souza

**Affiliations:** 1 Departamento de Energía y Mecánica, Carrera de Petroquímica, Universidad de las Fuerzas Armadas—ESPE, Sangolquí EC170501, Ecuador; jacordova8@espe.edu.ec; 2 Escola de Química, Universidade Federal do Rio de Janeiro (UFRJ), Centro de Tecnologia, Bloco E, Sala 206, CEP, Rio de Janeiro 21941-909, RJ, Brazil; mmattos@eq.ufrj.br

**Keywords:** double perovskite, LaNi_0.5_Ti_0.5_O_3_, La_2_NiTiO_6_, nanoparticles

## Abstract

In the present work, LaNi_0.5_Ti_0.5_O_3_ and La_2_NiTiO_6_ nanoparticles were synthesized by the modified Pechini method. LaNi_0.5_Ti_0.5_O_3_ was calcined at 1073 K for 17 h or 100 h, while La_2_NiTiO_6_ was calcined at 1273 K for 135 h. The double perovskite calcined at 1073 K for 17 h presented orthorhombic symmetry with *Pbnm* space group, mean particle size was 31.9 ± 1 nm, random ordering of Ni^2+^ and Ti^4+^ cations, Néel temperature close to 15 K, and magnetic moment of 1.29 $\mu_{B}$. By increasing the calcination time, this material showed the same symmetry and space group, a mean particle size of 50.7 ± 2 nm, short-range ordering of Ni^2+^ and Ti^4+^ cations, Néel temperature around 12 K, and magnetic moment of 0.96 $\mu_{B}$. La_2_NiTiO_6_ presented a monoclinic crystal structure, with *P*2_1_/*n* space group, mean particle size of 80.0 ± 5 nm, rock salt ordering of Ni^2+^ and Ti^4+^, Néel temperature of approximately 23 K, and magnetic moment of 2.75 $\mu_{B}$.

## 1. Introduction

Perovskites are materials represented by the formula ABX_3_. A cation can be an alkali, alkali earth, or lanthanide metal, B cations are transition metals, and X can be oxygen or a halide [1]. The structure can be illustrated by octahedra layers, formed by octahedra sharing vertices, in which X atoms are found. The B cations occupy 6-octahedrally coordinated sites in the octahedra center, while A cations are located in dodecahedral coordinated sites. These A cations are placed in the cavities between octahedra. Double perovskites are mixed oxides, which can be formed when B cations are replaced by B′ cations, including the equimolar substitution. There are three B-cation sublattice types known for double perovskites: random, rock salt, and layered [2]. The rock salt and layered double perovskites show ordered arrangement.

The multiple properties observed in perovskites make these materials attractive for electrochemical [3], electronic [4], spintronic [5], and catalytic applications. For example, the reforming of methane, which includes partial oxidation, steam reforming, and CO_2_ reforming, has been carried out using catalysts derived from perovskites [6]. The reforming of methane is employed to produce H_2_, which can be used for naphtha reforming and ammonia production.

Nanoparticles can be used as nanocatalyst precursors for hydrogen production [7]. Nanocatalysts would decrease the cost of Fischer–Tropsch synthesis or enable competitive biofuel production compared to crude oil [8].

Nanoparticles of La, Ti, and Ni-based perovskite were used as catalysts for the steam reforming of methane [9,10]. These materials present random [11] or rock salt [12,13,14] B and B′ cation ordering.

La, Ni, and Ti-containing perovskites have been prepared by various synthesis methods. Rodríguez et al. [11] synthesized the LaNi_1-x_Ti_x_O_3_ (0 ≤ x ≤ 0.5) system using the liquid mix technique, with synthesis temperature ranging from 723 to 1073 K, for several days. This same group synthesized La_2_MTiO_6_ (M = Ni, Co) compounds, using the aforementioned preparation method, and final thermal treatment in the range of 723 to 1173 K [12]. In these last two cases, the particle size was not reported. Yang et al. [15] synthesized the LaNi_1-x_Ti_x_O_3_ perovskites using the amorphous citrate decomposition method, with final heat treatment ranging between 973 to 1273 K, for 10 h. In this last case, LaNi_0.5_Ti_0.5_O_3_ was used as an acetone sensor. Pérez-Flores et al. [13,16] synthesized La_2−x_Sr_x_NiTiO_6-__δ_ (0 ≤ x ≤ 0.5) materials using a final temperature treatment of 1773 K, for 24 h, with an average crystallite size of approximately 8 nm. Moreover, this same group reported the synthesis of the La_2−x_NiTiO_6_ (0 ≤ x < 0.2) perovskites [17], along with La_2_NiTiO_6_ [18]. For the last double perovskite, the particle size ranged from 40 to 80 nm. Yang et al. [14] synthesized La_2_Ni(Mn_1−x_Ti_x_)O_6_ materials using the solid-state reaction, employing a final thermal treatment at 1773 K, for 3 h. LaNi_0.5_Ti_0.5_O_3_, LaNi_0.5_Ti_0.45_Co_0.05_O_3_, and LaNi_0.45_Co_0.05_Ti_0.5_O_3_ synthesized by the modified Pechini method, coupled to a final heat treatment of 1073 K, for 17 h, with crystallite sizes of 27.1 nm, 35.8 nm, and 32.7 nm, respectively, were reported in previous works [9,10,19].

Furthermore, no works of LaNi_0.5_Ti_0.5_O_3_ and La_2_NiTiO_6_ double perovskite nanoparticles were reported in the literature, using thermal treatments with final temperatures lower than those used in our work.

This article aims to study the structural and magnetic properties of LaNi_0.5_Ti_0.5_O_3_ and La_2_NiTiO_6_ double perovskite nanoparticles, synthesized with a calcination temperature lower than the corresponding thermal conditions reported for the same double perovskites.

## 2. Materials and Methods

### 2.1. Synthesis

Nanoparticles of La, Ni, and Ti-containing double perovskites were prepared by the modified Pechini method [20]. Metal to the citric acid molar ratio of 1:2 and citric acid to ethylene glycol molar ratio of 1:4 were used for the synthesis. Adequate amounts of precursors (lanthanum nitrate hexahydrate (La(NiO_3_)_3_6H_2_O, 99.5%), nickel nitrate hexahydrate (Ni(NiO_3_)_2_6H_2_O, 97%), and titanium isopropoxide (C_12_H_28_O_4_Ti, 97%) from Sigma-Aldrich) were dissolved in water. For the case of titanium, it was dissolved in 30 mL of nitric acid solution (3.4 mol L^−1^) after isopropoxide evaporation. Initially, titanium citrate was prepared by heating titanium solution to 333 K for citric acid addition. Next, ammonium hydroxide was added to the titanium citrate solution in a volume close to nitric acid. Then, this last solution was aged overnight at room temperature. Lanthanum and nickel citrates were prepared using the methodology of titanium citrate preparation, except the ammonium hydroxide addition. Citrates were mixed at room temperature and heated up to 363 K with the addition of ethylene glycol to form a polyester solution. This final solution was aged similarly to metal citrate solution, with subsequent evaporation at 333 K, calcined at 513 K for 1 h, and 723 K for 4 h, using a heating rate of 2 K min^−1^, milled in an agate mortar, obtaining the precursor.

Sample 1 was obtained by precursor calcination in air at 1073 K for 17 h. Sample 2 was synthesized by subsequent milling in agate mortar and calcination of Sample 1 at 1073 K for 100 h. Finally, Sample 3 was prepared by calcination of the precursor in air at 1173 K for 17 h, milled in an agate mortar, and calcined again at 1273 K for 135 h.

### 2.2. Characterization

The chemical composition of the samples was determined by X-ray fluorescence (XRF) using a Rigaku Primini spectrometer equipped with a Pd X-ray tube operating at 50 W (40 kV, 1.25 mA) and a ZSX software package. A 200 mg sample mass was placed in a polyethylene sample cup, covered with a thin polypropylene film, and then fixed with a ring.

X-ray diffraction (XRD) measurements were performed in a Rigaku Miniflex II X-ray diffractometer equipped with a graphite monochromator, using CuKα radiation (30 kV and 15 mA). XRD patterns of as-prepared samples were recorded over the 2θ range of 5–90°, step size and counting time per step equal to 0.02° and 6 s. A quartz sample holder was employed, on which a sample layer close to 300 mg was placed.

Rietveld method of X-ray diffraction patterns was performed using Fullprof Suite Program 3.0 [21]. The structure models of LaNi_0.5_Ti_0.5_O_3_ (ICSD: 88851, [22]) and La_2_NiTiO_6_ (ICSD: 95977, [22]) double perovskites were employed for structure refinement of calcined materials at 1073 K (Sample 1 and Sample 2), and 1273 K (Sample 3), respectively. The background was defined with a 4th-order polynomial and refined simultaneously with the scale factor, unit cell parameters, peak shape parameters of the pseudo-Voigt function, atomic coordinates, together with the sample displacement, Sycos. For convergence achievement, xO2 from Sample 1 and zO1, and zO3 from Sample 3 were not refined. Sycos is the ratio between a parameter accounting for the sample displacement error, in degrees, and cosine of θ angle, where θ is measured in radians [23]. The isotropic displacement parameter was maintained at the value equal to 0.5 Å^2^, as suggested for atoms in a metal oxide [24]. The fraction of site occupancy was not refined and was maintained at the value reported by ICSD [22] (see columns five and six from Table S1), except that of Ni^2+^ and Ti^4+^ from Sample 3.

Crystallite size was determined by the Scherrer equation. This equation was applied to (112) peak for Sample 1 and Sample 2, or $\left(\bar{1}12\right)\;$peak for Sample 3, found at 2θ equal to 32.18°.

Micrographs were obtained from a scanning electron microscope (SEM, Model Quanta^TM^ 450 FEG, FEI Company, Hillsboro, OR, USA) operating with an accelerating voltage of 20 kV. Before the analysis, the samples were coated by sputtering with an Au-containing film. Micrographs were analyzed with ImageJ version 1.52 v program [25]. Mean particle size was obtained using 100 particles.

DC magnetic measurements as a function of temperature, in the zero-field-cooled process, applying a magnetic field of 100 Oe, were performed using an MPMS 3 SQUID magnetometer in the temperature range 2–250 K (Sample 1), and Cryogenic SX-600 SQUID magnetometer in the temperature range 2–120 K (Sample 2 and Sample 3).

## 3. Results and Discussions

Table S1 shows the chemical composition of the samples determined by XRF. Compared to the corresponding theoretical counterparts, these values present minor variations. These differences can be attributed to equipment measurement errors and confirm the accuracy of the preparation method.

**Figure 1:**
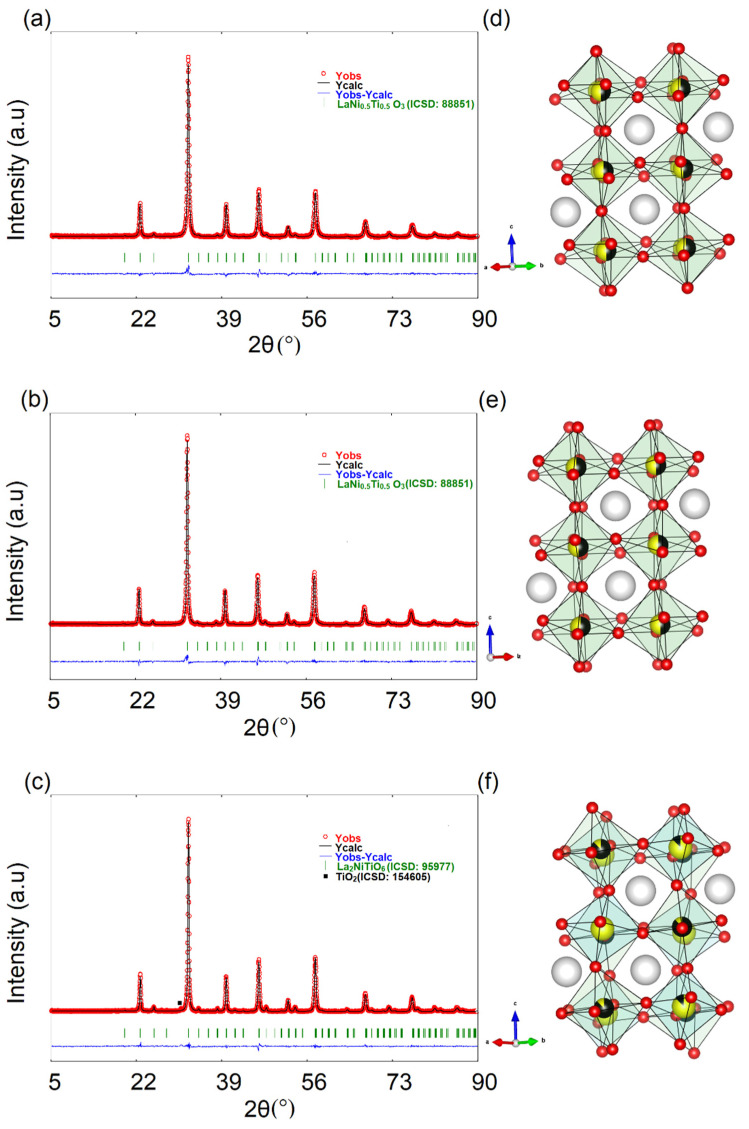
Observed (red symbols), calculated (black line) and difference (blue line) X-ray diffraction profiles of (**a**) Sample 1, (**b**) Sample 2 and (**c**) Sample 3. Crystal structure of (**d**) Sample 1, (**e**) Sample 2, and (**f**) Sample 3. (White, black, yellow, and red spheres denote La, Ni, Ti, and O atoms, respectively).

Figure 1 depicts the observed, calculated, and difference XRD patterns of the as-prepared double perovskites. For the case of Sample 1 and Sample 2, a single phase was obtained without any impurity. Sample 3 was composed of La_2_NiTiO_6_ and TiO_2_, with phase percentages equal to 97.28 % and 2.72 %, respectively. These values can be verified in Figure S1 and Table S2 of the Supplementary Material. Thus, Sample 3 was almost a single phase. The structural parameters and R-factor values are reported in Table S1, while the bond distance and bond angles are reported in Table S3. The unit cell parameters, atomic coordinates, the fraction of site occupancy values (Sample 3) and bond distance, along bond angles from the as-prepared double perovskites, are close to the values reported by ICSD [22].

Moreover, R-factor values from the Rietveld refinement are expected for the assumed experimental conditions. Thus, Sample 1 and Sample 2 have orthorhombic symmetry with the *Pbnm* space group. Further, Supplementary Material Figure S2 presents the Rietveld refinement of Sample 3, using *Pbnm* space group (ICSD: 88851, [22]). Furthermore, Table S4 shows the respective Rietveld refinement results. The Bragg factor from this Table is higher than that obtained from the *P*2_1_/*n* space group (3.89 (Table S4) vs. 3.14 (Table S1)). The same tendency was observed for the case of the chi-square value (1.82 (Table S4) vs. 1.64 (Table S1)). Therefore, Sample 3 shows monoclinic symmetry, with *P*2_1_/*n* space group. The symmetry and space group are according to that reported in the literature for the LaNi_0.5_Ti_0.5_O_3_ [11] (Sample 1 and Sample 2) and La_2_NiTiO_6_ [12,13,14] (Sample 3). The Glazer notation for as-synthesized materials is a^−^a^−^c^+^, as reported for the case of the orthorhombic [19] and monoclinic [26] symmetries. Verification of the crystal structure was performed from the indexation of each XRD pattern, using McMaille software [27], with calculations reported in Figures S3–S8 of the Supplementary Material. Each double perovskite crystal structure is indicated in Figure 1, which was drawn using VESTA software [28]. 

**Figure 2:**
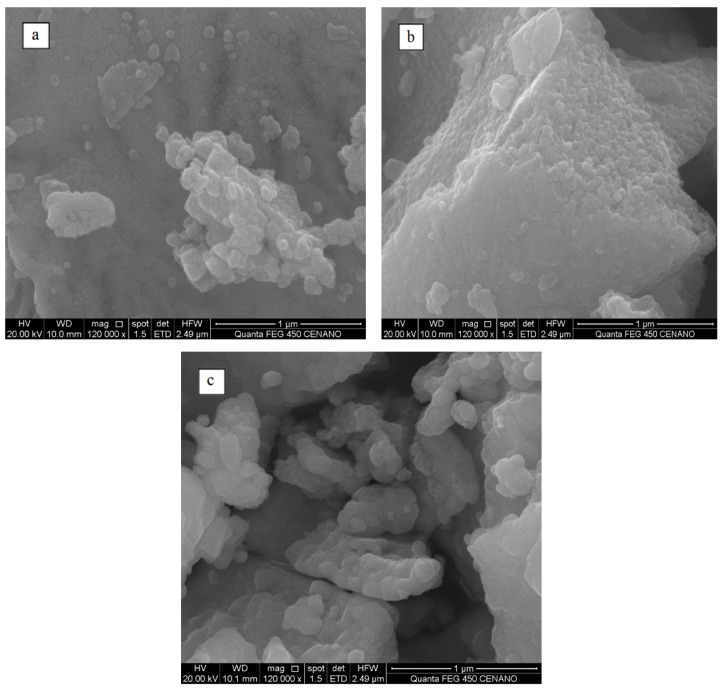
FEG-SEM images of (**a**) Sample 1, (**b**) Sample 2, and (**c**) Sample 3.

FEG-SEM images of the as-synthesized materials are shown in Figure 2. Sample 1 and Sample 2 materials are composed of agglomerated nanoparticles (Figure 2a,b). Aggregated particles at the nanoscale form Sample 3 are shown in Figure 2c. The mean particle sizes of Sample 1, Sample 2, and Sample 3 were equal to 31.9 ± 1 nm, 50.7 ± 2 nm, and 80.0 ± 5 nm, respectively. The measured particle sizes are in accordance with the respective crystallite sizes (Sample 1: 27.1 nm; Sample 2: 32.4 nm; Sample 3: 39.8 nm; Table S1).

**Figure 3:**
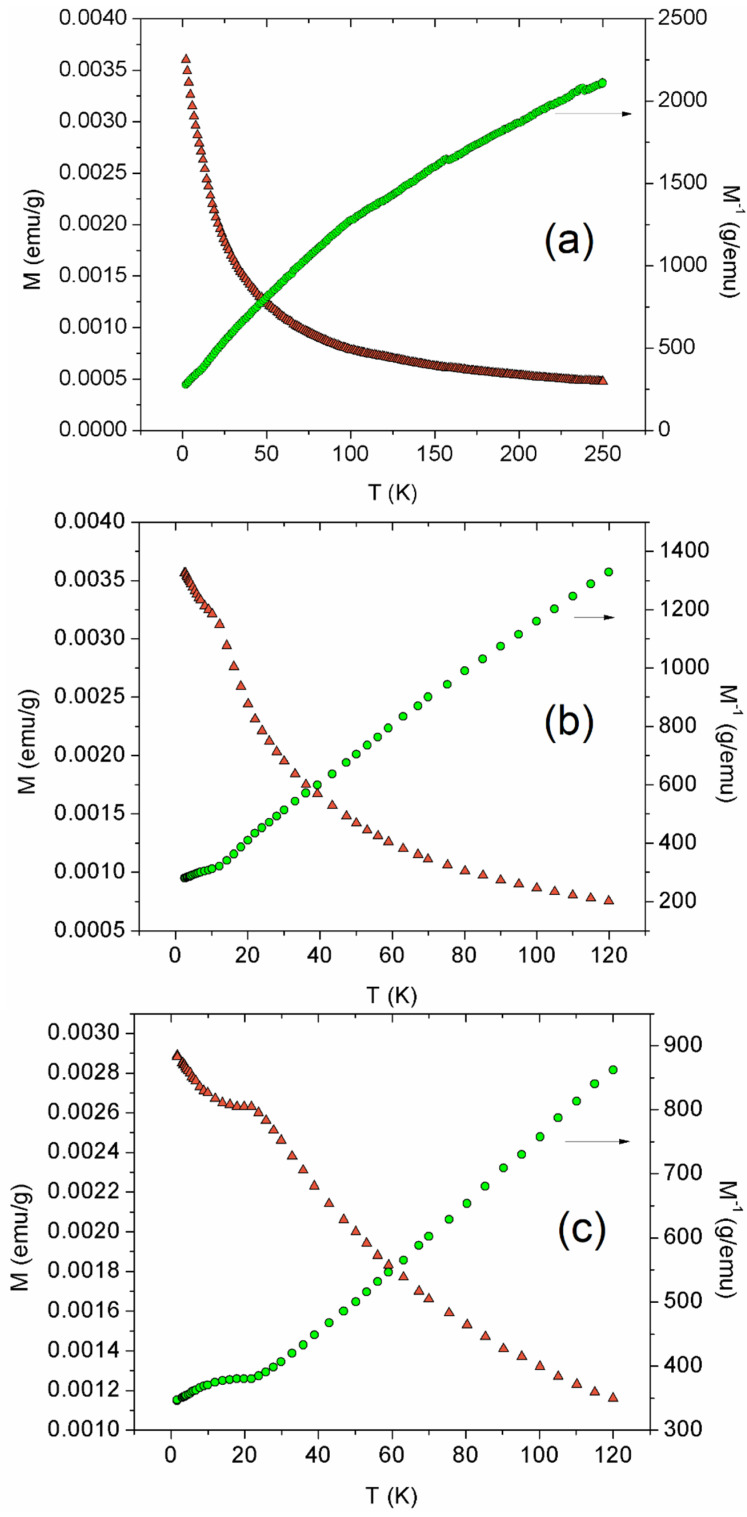
Variation of M along with M^−1^ vs. temperature for (**a**) Sample 1, (**b**) Sample 2, and (**c**) Sample 3.

Figure 3 shows the magnetization and the respective inverse as a function of temperature, under zero-field cooling conditions. 

$\chi_{m}T$ vs. T for Sample 1 is depicted in Supplementary Material Figure S9. Sample 1 shows Néel temperature around 15 K, as evidenced in Figure S9. This is the temperature at which a steep decrease in $\chi_{m}T\;$values occurs, as the sample is cooled during the analysis [29]. The evolution of $\chi_{m}T\;$with temperature is in agreement with that reported for the LaNi_0.5_Ti_0.5_O_3_ [11]. Sample 2 and Sample 3 present Néel temperature around 12 K and 23 K, respectively. This last value is close to the respective values found in the work of Pérez-Flores et al. [13] and Rodríguez et al. [12] (25 K), or presented by Yang et al. [14] (17 K). 

B and B′ cation ordering increases with Néel temperature, up to 25 K in the $\chi_{m}$ vs. T curve, as reported for the La_2−x_Sr_x_NiTiO_6-δ_ system [13]. Since Sample 1 did not show any peak in the M vs. T curve (Figure 3), a random arrangement of B and B′ cations is confirmed for this sample. The Néel temperature value for Sample 2 can be attributed to short-range ordering. B and B′ short-range cation ordering was shown by Pérez-Flores et al. [13] for the case of the La_1.85_Sr_0.15_NiTiO_6_ material, when compared to that observed for La_2_NiTiO_6_. Moreover, the rock salt ordering arrangement of B and B′ cations can be assigned to Sample 3. 

For the case of Sample 1 and Sample 3, B and B′ cation ordering relates to symmetry determined from X-ray diffraction, coupled to the Rietveld method. The Néel temperature value for Sample 3 is indicative of B and B′ cation ordering, with *P*2_1_/*n* space group [12,13,14]. On the other hand, the short-range order of Sample 2 fits adequately into the orthorhombic symmetry, as presented by Pérez-Flores et al. [13] for La_1.85_Sr_0.15_NiTiO_6_ perovskite.

The inverse values of the magnetic susceptibility were fitted using the Curie–Weiss law for the effective magnetic moment calculation. Curie constant, Weiss temperature, and the range used for the fitting are presented in Table 1. The magnetic moment for Sample 1 and Sample 2 (1.29 $\mu_{B}$, and 0.96 $\mu_{B}$, Table 1) are similar to the value found by Rodríguez et al. [11] (0.93 $\mu_{B}$) for the LaNi_0.5_Ti_0.5_O_3_. It is essential to point out that this analysis was redone for Sample 2, using Physical Property Measurement System (Quantum Design) equipment, in the range 2–40 K, along with a magnetic field equal to 1000 Oe, and presented in Figure S10 of the Supplementary Material.

Moreover, the effective magnetic moment for Sample 3 (2.75 $\mu_{B}$, Table 1) is in agreement with the values reported for La_2_NiTiO_6_ (3.12 $\mu_{B}$, [12]; 3.09(2) $\mu_{B}$, [13]).

## 4. Conclusions

Nanoparticles of LaNi_0.5_Ti_0.5_O_3_ and La_2_NiTiO_6_ were synthesized using the modified Pechini method. LaNi_0.5_Ti_0.5_O_3_ calcined at 1073 K for 17 h, showing orthorhombic symmetry with *Pbnm* space group, with mean particle size equal to 31.9 ± 1 nm, Néel temperature value around 15 K, and magnetic moment of 1.29 $\mu_{B}$. By changing 17 h to 100 h for the calcination time, this material presented the same crystal structure and space group, mean particle size equal to 50.7 ± 2 nm, Néel temperature value approximately 12 K, and magnetic moment of 0.96 $\mu_{B}$. On the other hand, La_2_NiTiO_6_ showed a monoclinic crystal structure, with *P*2_1_/*n* space group, mean particle size equal to 80.0 ± 5 nm, Néel temperature value around 23 K, and magnetic moment of 2.75 $\mu_{B}$. The Glazer notation a^-^a^-^c^+^ can represent these as-synthesized perovskites.

Random Ni^2+^ and Ti^4+^ cation ordering was verified for the LaNi_0.5_Ti_0.5_O_3_ sample synthesized with the shortest calcination time. Short-range ordering arrangement of these transition metals was assigned to LaNi_0.5_Ti_0.5_O_3,_ calcined for 100 h. In addition, the rock salt order of the Ni^2+^ and Ti^4+^ cations was confirmed for the La_2_NiTiO_6_.

## Figures and Tables

**Table 1:** Weiss temperature (θ), Curie constant (C), and the effective magnetic moments ($\mu_{eff}$) for Sample 1, Sample 2, and Sample 3.

Sample	ΔT (K) ^a^	θ	C (emu K Oe^−1^ mol^−1^)	$\mu_{eff}$ $\;(\mu_{B})$
1	14–40	−15	0.207456	1.29
2	14–40	−12	0.114417	0.96
3	33–120	−23	0.944706	2.75

^a^ Temperature range used for estimation of $\mu_{eff}$

## Data Availability

Not applicable.

## Supplementary Materials

The following supporting information can be downloaded at: https://www.mdpi.com/article/10.3390/ma15072411/s1, Figure S1: Observed (red symbols), calculated (black line), and difference (blue line) XRD profiles of Sample 3, Figure S2: Observed (red symbols), calculated (black line) and difference (blue line) X-ray diffraction profile of Sample 3 using *Pbnm* space group (ICSD: 88851, [1]), Figure S3: Both unit-cell parameters and corrected observed 2-theta values based on the Rietveld Refinement of XRD data of Sample 1 to perform the indexation of the corresponding XRD pattern, Figure S4: Both unit-cell parameters and corrected observed 2-theta values based on the Rietveld Refinement of XRD data of Sample 2 to perform the indexation of the corresponding XRD pattern, Figure S5: Both unit-cell parameters and corrected observed 2-theta values based on the Rietveld Refinement of XRD data of Sample 3 to perform the indexation of the corresponding XRD pattern, Figure S6: Unit-cell parameters, the corrected observed and calculated peak positions, and the respective difference obtained from the McMaille software for the case of Sample 1, Figure S7: Unit-cell parameters, the corrected observed and calculated peak positions, and the respective difference obtained from the McMaille software for the case of Sample 2, Figure S8: Unit-cell parameters, the corrected observed and calculated peak positions, and the respective difference obtained from the McMaille software for the case of Sample 3, Figure S9: $\chi_{m}T$ vs. T curve showing the Néel temperature for Sample 1, Figure S10: Variation of M along with M^−1^ vs. temperature for Sample 2 from 2 to 40 K; Table S1: Structural parameters, average crystallite size, together with conventional discrepancy factors from Rietveld refinement of XRD data for Sample 1, Sample 2, and Sample 3 [30], Table S2: Structural parameters, results of phase analysis together with conventional discrepancy factors from Rietveld refinement of XRD pattern for Sample 3, Table S3: Bond distances and bond angles for Sample 1, Sample 2, and Sample 3, Table S4: Structural parameters, average crystallite size, together with conventional discrepancy factors from Rietveld refinement of XRD data for Sample 3.
