# Supplementary material for: Synthesis and Characterization of LaNi0.5Ti0.5O3 and La2NiTiO6 Double Perovskite Nanoparticles
Source: Materials (Basel). 2022 Mar 25;15(7):2411. doi: 10.3390/ma15072411 (PMC8999483; doi:10.3390/ma15072411)
Supplement: Supplementary file 1 [file materials-15-02411-s001.zip › materials-1631572-supplementary.pdf]

Supplementary Material

Synthesis and Characterization of $LaNi_{0.5}Ti_{0.5}O_3$ and La_2NiTiO_6 Double Perovskite Nanoparticles

José Córdova-Calderón [1], Pablo V. Tuza [1,*] and Mariana M. V. M. Souza [2]

[1] Departamento de Energía y Mecánica, Carrera de Petroquímica, Universidad de las Fuerzas Armadas—ESPE, EC170501 Sangolquí, Ecuador; jacordova8@espe.edu.ec

[2] Escola de Química, Universidade Federal do Rio de Janeiro (UFRJ), Centro de Tecnologia, Bloco E, Sala 206, CEP, Rio de Janeiro 21941–909, RJ, Brazil; mmattos@eq.ufrj.br

* Correspondence: pvtuza@espe.edu.ec; Tel.: +593-32810206 (ext. 4303)

Table S1. Structural parameters, average crystallite size, together with conventional discrepancy factors from Rietveld refinement of XRD data for Sample 1, Sample 2, and Sample 3.

	**Sample 1**^{a,c}	**Sample 2**^{a,d}	**Sample 3**^{a,e}	**$LaNi_{0.5}Ti_{0.5}O_3$ (ICSD: 88851)**^{b}	**La_2NiTiO_6 (ICSD: 95977)**^{b}
La_2O_3 (wt%)	66.67	67.55	66.75		
NiO (wt%)	16.79	16.41	16.58		
TiO_2 (wt%)	16.54	16.04	16.67		
Sycos (°)	−0.00685	0.00454	0.06828		
Average crystallite size (nm)^{f}	27.1	32.4	39.8		
X-ray density (g cm^{-3})	6.643	6.640	6.681	6.631	6.605
Space group^{g}	*Pbnm*	*Pbnm*	$P2_1/n$	*Pbnm*	$P2_1/n$
a/(Å)	5.540(2)	5.539(2)	5.541(1)	5.517	5.5545
b/(Å)	5.533(2)	5.538(1)	5.534(1)	5.551	5.5512
c/(Å)	7.834(3)	7.832(3)	7.823(2)	7.856	7.8341
β/(°)			90.053(35)		90.08
V(Å^3)	240.2(1)	240.3(1)	239.9(1)	240.6	241.6
A Position	4c	4c	4e	4c	4e
x	−0.00456(41)	0.00378(82)	0.50438(75)	-0.0048	0.5080
y	0.01976(27)	0.02238(21)	0.52603(19)	0.0206	0.5303
z	0.25	0.25	0.24973(172)	0.25	0.2586
Occ	0.5	0.5	1	0.5	1
B' Position	4b	4b	2d	4b	2d
Occ^{h}	0.25	0.25	0.433(1)/ 0.077(2)	0.25	0.43/0.07
B'' Position	4b	4b	2c	4b	2c
Occ^{i}	0.25	0.25	0.433(1)/ 0.077(2)	0.25	0.43/0.07
O(1) Position	8d	8d	4e	8d	4e
x	0.25306(317)	0.25763(292)	0.25902(734)	0.281	0.2286
y	0.29214(227)	0.28780(231)	0.22762(541)	0.249	0.2173
z	0.04430(117)	0.04495(173)	-0.02690	0.036	−0.0269
Occ	1	1	1	1	1
O(2) Position	4c	4c	4e	4c	4e
x	0.02	-0.04730(325)	0.29993(869)	0.02	0.2945
y	0.478(2)	0.48236(184)	0.69223(750)	0.499	0.7106
z	0.25	0.25	−0.03767(437)	0.25	−0.0376
Occ	0.5	0.5	1	0.5	1
O(3) Position			4e		4e
x			0.42628(714)		0.4266
y			0.97984(193)		0.9928
z			0.24110		0.2411

Occ			1	1
Chi-square (χ^2)	1.68	1.59	1.64	
Rp/Rwp/R_B	12.7/14.6/4.3	12.1/13.9/3.7	11/14.1/3.14	

[a]Theoretical chemical composition for La_2O_3 (wt%) / NiO (wt%) / TiO_2 (wt%): 67.82 % / 15.55 % / 16.63 %.[b]Reference: ICSD [1]. [c]F(20) = 20.04 (0.0082, 122) [2]. [d]F(20) = 29.82 (0.0058, 115) [2]. [e]F(20) = 16.73 (0.0137, 98) [2]. [f]The (1$\bar{1}$2) and (112) planes were selected for the Sample 3 and the other perovskites, respectively. [g]$P2_1/n$: 4e(x y z), 2d(0.5 0 0), 2c(0 0.5 0). [g]*Pbnm*: 4c(x y 0.25), 4b(0.5 0 0), 8d(x y z). $P2_1/n$: Occ[h] is mainly Ti, and Occ[i] is mostly Ni; *Pbnm*: Occ[h] and Occ[i] are Occ Ni/Ti.

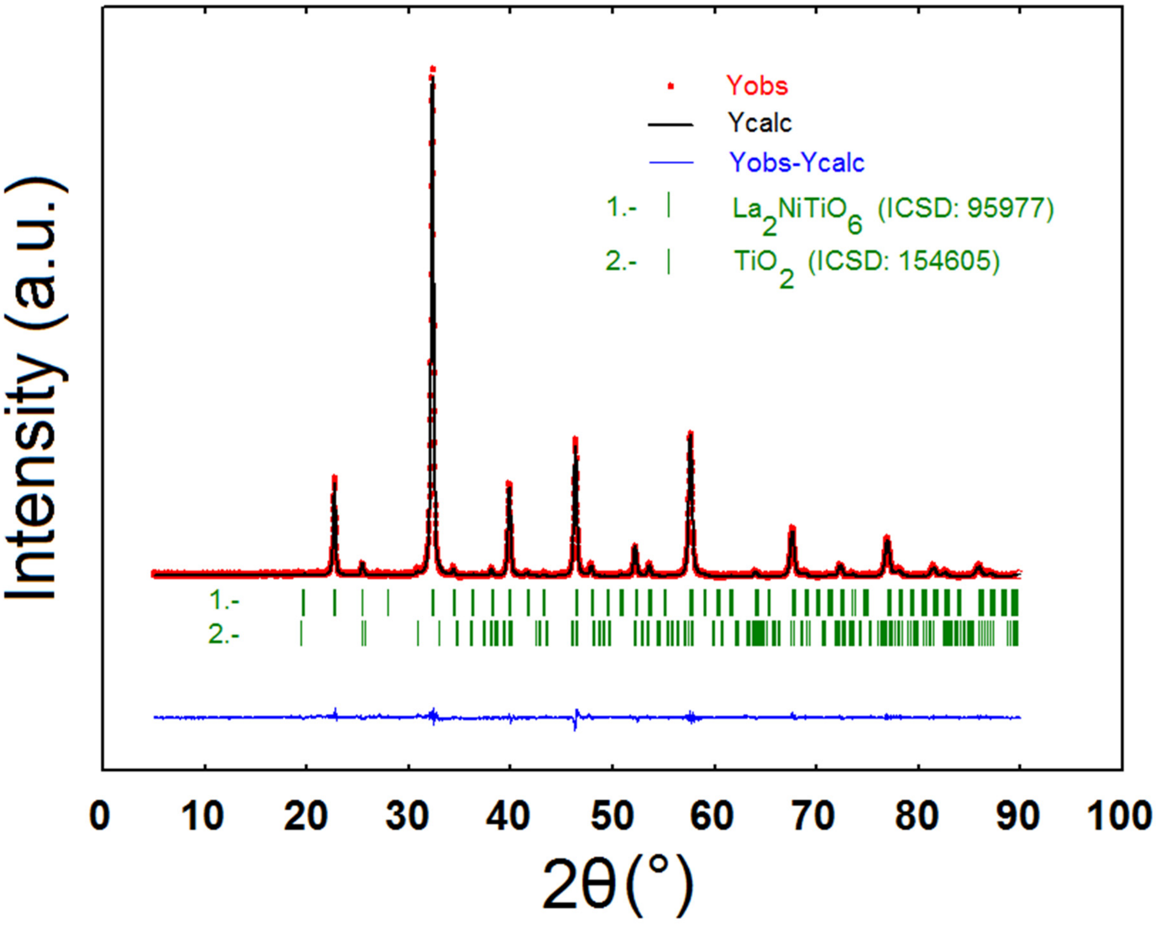

Figure S1. Observed (red symbols), calculated (black line), and difference (blue line) XRD profiles of Sample 3.

Table S2. Structural parameters, results of phase analysis together with conventional discrepancy factors from Rietveld refinement of XRD pattern for Sample 3. .

Parameter	Sample 3	ICSD [1]
**La_2NiTiO_6[a]**		
a	5.541(1)	5.5545
b	5.534(1)	5.5512
c	7.823(1)	7.8341
β(°)	90.06(3)	90.08
La: x	0.50494(76)	0.508
y	0.52553(19)	0.5303
z	0.25141(137)	0.2586
B(Å^2)	0.5	0.53
Occ	1	1
Ni1: x	0.5	0.5
y	0	0
z	0	0

B(Å^2)	0.5	1.8
Occ	0.076(2)	0.07
Ti1: x	0.5	0.5
y	0	0
z	0	0
B(Å^2)	0.5	1.8
Occ	0.434(1)	0.43
Ni2: x	0	0
y	0.5	0.5
z	0	0
B(Å^2)	0.5	1.39
Occ	0.434(1)	0.43
Ti2: x	0	0
y	0.5	0.5
z	0	0
B(Å^2)	0.5	1.39
Occ	0.076(2)	0.07
O1: x	0.25699(594)	0.2286
y	0.22996(535)	0.2173
z	−0.0269	−0.0269
B(Å^2)	0.5	2.5
Occ	1	1
O2: x	0.31358(731)	0.2945
y	0.69316(641)	0.7106
z	−0.04578(408)	−0.0376
B(Å^2)	0.5	0.66
Occ	1	1
O3: x	0.44120(644)	0.4266
y	0.98731(177)	0.9928
z	0.24110	0.2411
B(Å^2)	0.5	0.69
Occ	1	1
Scale factor(1x10^4)	3.09024 (950)	
Composition (wt.%)	97.28(0.42)	
*R*bragg (%)	3.07	
**TiO_2**^{b}		
a	9.167(2)	9.18
b	5.438(1)	5.43
c	5.1649(3)	5.164
Ti: x	0.1289	0.1289
y	0.0992	0.0992
z	0.8628	0.8628
B(Å^2)	0.5	0.93
Occ	1	1
O1: x	0.0098	0.0098
y	0.148	0.148
z	0.1838	0.1838
B(Å^2)	0.5	1.08
Occ	1	1
O2: x	0.2312	0.2312
y	0.1116	0.1116
z	0.5362	0.5362
B(Å^2)	0.5	1.13
Occ	1	1
Scale factor (1x10^5)	1.2182(70)	
Composition (wt.%)	2.72(0.02)	
*R*bragg (%)	17.2	
Rp (%)	11	

Rwp (%)	14
$\chi 2$	1.63

a: La_2NiTiO_6(ICSD: 95977). b: TiO_2(ICSD: 154605).

Table S3. Bond distances and bond angles for Sample 1, Sample 2, and Sample 3.

	Sample 1	Sample 2	Sample 3	$LaNi_{0.5}Ti_{0.5}O_3$ (ICSD: 88851)[a]	La_2NiTiO_6 (ICSD: 95977)[a]
Ni/Ti—O1	2.146(14) × 2<br>1.847(14) × 2	2.113(14) × 2<br>1.883(15) × 2		2.10348 × 2<br>1.85749 × 2	
Ni/Ti —O2	1.9654(10) × 2	1.978(2) × 2		1.96711 × 2	
Ti—O1			1.85(3) × 2		1.94195 × 2
Ti—O2			2.05(5) × 2		1.99239 × 2
Ti —O3			1.934(8) × 2		1.93327 × 2
Ni—O1			2.09(3) × 2		2.02983 × 2
Ni—O2			2.00(5) × 2		2.03241 × 2
Ni—O3			2.069(8) × 2		2.06865 × 2
Ni/Ti —O1— Ni/Ti	157.4(6)	157.1(6)	167.3(14)	162.1	162.668
Ni/Ti —O2— Ni/Ti	170.4(1)	163.84 (13)	150.4(19)	173.6	154.614
Ni/Ti —O3— Ni/Ti			155.5(3)		156.345

[a]Reference: ICSD [1].

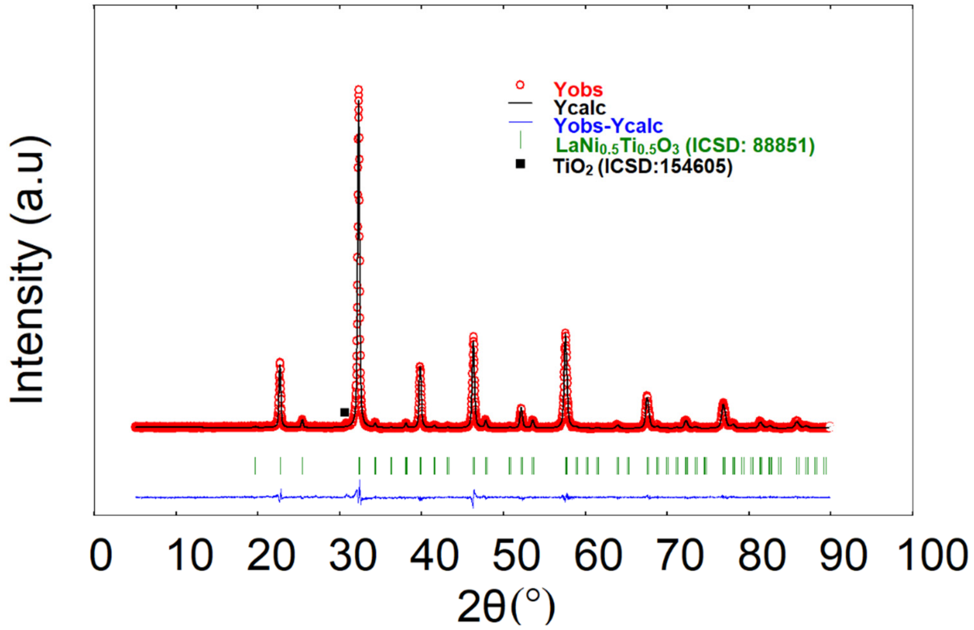

Figure S2. Observed (red symbols), calculated (black line) and difference (blue line) X-ray diffraction profile of Sample 3 using *Pbnm* space group (ICSD: 88851, [1]).

Table S4. Structural parameters, average crystallite size, together with conventional discrepancy factors from Rietveld refinement of XRD data for Sample 3.

	**Sample 3**[a]	**$LaNi_{0.5}Ti_{0.5}O_3$ (ICSD: 88851)**[b]
La_2O_3 (wt%)	66.75	
NiO (wt%)	16.58	
TiO_2 (wt%)	16.67	
Sycos (°)	0.12418	
Average crystallite size (nm)[c]	39.8	
X-ray density (g cm^{-3})	6.622	6.631
Space group[d]	*Pbnm*	*Pbnm*
a/(Å)	5.5448(14)	5.517
b/(Å)	5.5428(7)	5.551
c/(Å)	7.8391(19)	7.856
V(Å^3)	240.9(1)	240.6
A Position	4c	4c
x	0.0052(6)	−0.0048
y	0.02633(18)	0.0206
z	0.25000	0.25
Occ	0.5	0.5
B' Position	4b	4b
Occ Ni/Ti	0.25	0.25
B'' Position	4b	4b
Occ Ni/Ti	0.25	0.25
O(1) Position	8d	8d
x	0.266(3)	0.281
y	0.288(2)	0.249
z	0.0463(16)	0.036
Occ	1	1
O(2) Position	4c	4c
x	−0.056(3)	0.02
y	0.4821(17)	0.499
z	0.25	0.25
Occ	0.5	0.5
O(3) Position		
x		
y		
z		
Occ		
Chi-square (χ^2)	1.82	
Rp/Rwp/R_B	12.3/14.8/3.89	

[a]Theoretical chemical composition for La_2O_3 (wt%) / NiO (wt%) / TiO_2 (wt%): 67.82 % / 15.55 % / 16.63 %. [b]Reference: ICSD [1]. [c]It was selected the (112) plane. [d]*Pbnm*: 4c(x y 0.25), 4b(0.5 0 0), 8d(x y z).

```
! Wavelength, zeropoint, Ngrid
 1.540600   0.0000 0
! Codes for symmetry
 0 0 0 1 0 0
! W, Nind
0.300  3
!Pmin, Pmax, Vmin, Vmax, Rmin, Rmax, Rmaxref
  -2.  50.  8.  125000.  0.05 0.15 0.50
5.540,5.540,5.5334,5.5334,7.834,7.834,90,90,90,90,90,90
! Ntests, Nruns
 -100 20
!   2-theta    Intensity
   22.729     1165.66077
   25.483     166.01009
   32.332     6113.91406
   34.264     134.48129
   37.955     124.73923
   39.884     1151.93445
   41.602     113.57584
   43.135     112.58904
   46.398     1674.59241
   47.800     171.24411
   52.237     371.15247
   57.677     1591.25061
   67.655     570.33246
   72.260     193.13924
   72.433     182.96774
   77.018     469.38516
   78.087     132.52876
   78.214     137.56558
   81.350     161.02144
   81.456     160.8553
   85.987     173.49216
```

Figure S3. Both unit-cell parameters and corrected observed 2-theta values based on the Rietveld Refinement of XRD data of the Sample 1 to perform the indexation of the corresponding XRD pattern.

```
! Wavelength, zeropoint, Ngrid
 1.540600   0.0000 0
! Codes for symmetry
 0 0 0 1 0 0
! W, Nind
0.300  3
!Pmin, Pmax, Vmin, Vmax, Rmin, Rmax, Rmaxref
  -2. 50. 8. 125000. 0.05 0.15 0.50
5.539,5.539,5.538,5.538,7.832,7.832,90,90,90,90,90,90
! Ntests, Nruns
 -100 20
!   2-theta   Intensity
   19.491     111.19807
   22.721     1377.63684
   25.372     168.81578
   27.908     102.65396
   32.329     7107.37012
   34.405     127.2098
   38.002     140.27457
   38.125     140.02853
   39.866     1307.08606
   41.621     107.36599
   46.396     1903.33923
   47.747     167.05431
   47.87     175.30615
   50.784     96.97334
   52.206     436.61438
   53.68     152.98846
   57.655     1882.16492
   64.005     127.13898
   67.657     709.97998
   68.749     121.28933
   71.118     91.8567
   72.355     215.15233
   77.005     586.34283
   81.562     170.39136
   83.776     80.01309
   85.897     183.95049
```

Figure S4. Both unit-cell parameters and corrected observed 2-theta values based on the Rietveld Refinement of XRD data of the Sample 2 to perform the indexation of the corresponding XRD pattern.

```
! Wavelength, zeropoint, Ngrid
 1.540600   0.0000 0
! Codes for symmetry
 0 0 0 0 1 0
! W, Nind
0.300  3
!Pmin, Pmax, Vmin, Vmax, Rmin, Rmax, Rmaxref
  -2. 50. 8. 125000. 0.05 0.15 0.50
5.541,5.541,5.534,5.534,7.823,7.832,90,90,90.053,90.053,90,90
! Ntests, Nruns
 -100 20
!   2-theta   Intensity
   19.646     98.55988
   22.755     1594.68848
   25.448     252.16383
   27.835     84.39839
   32.352     7990.29395
   34.397     182.82043
   38.139     180.88072
   39.875     1483.26709
   41.56     110.71102
   41.648     112.81496
   43.223     98.07073
   46.389     2156.59546
   47.872     219.73148
   49.311     67.40535
   50.846     102.5596
   52.214     515.2486
   53.581     237.08159
   57.629     2281.96021
   60.398     77.94698
   61.405     79.21719
   61.638     64.80818
   63.942     145.94818
   67.594     811.90674
   68.822     106.70824
   70.053     68.18274
   72.284     223.97472
   73.432     100.66191
   76.924     590.33081
   78.027     173.95406
   78.275     145.45277
   81.38     208.30444
   81.459     192.9984
   82.484     120.6182
   82.603     136.0921
   82.725     102.89496
   85.819     212.56348
   85.964     225.44009
   86.918     107.56834
   87.033     108.12737
   87.233     103.72082
```

Figure S5. Both unit-cell parameters and corrected observed 2-theta values based on the Rietveld Refinement of XRD data of the Sample 3 to perform the indexation of the corresponding XRD pattern.

FINAL VALUES : (STANDARD DEVIATIONS : 2nd LINE)

ZERO	LAMBDA	A	B	C	ALPHA	BETA	GAMMA
0.000	1.5406	5.5279	5.5345	7.8414	90.000	90.000	90.000
0.011	0.0000	0.0010	0.0010	0.0014	0.000	0.000	0.000
RECIPROCAL CELL :		0.18090	0.18068	0.12753	90.000	90.000	90.000
VOLUME (A**3) :		239.904					

H	K	L	TH(OBS)	TH-ZERO	TH(CALC)	DIFF.
1	1	0	22.729	22.729	22.717	0.012
0	2	0	32.332	32.332	32.325	0.007
0	0	3	34.264	34.264	34.280	-0.016
2	0	2	39.884	39.884	39.874	0.010
2	2	0	46.398	46.398	46.394	0.003
0	2	3	47.800	47.800	47.830	-0.031
1	3	0	52.237	52.237	52.231	0.006
3	1	2	57.677	57.677	57.687	-0.010
0	4	0	67.655	67.655	67.659	-0.004
0	0	6	72.260	72.260	72.230	0.030
3	3	0	72.433	72.433	72.434	-0.001
2	4	0	77.018	77.018	77.010	0.008
0	4	3	78.087	78.087	78.088	-0.001
4	2	1	78.214	78.214	78.207	0.006
0	2	6	81.350	81.350	81.360	-0.010
4	0	4	85.987	85.987	85.995	-0.008

M(20) = 31.06
F(20) = 20.04 (0.0082, 122)

Final Rp on the .prf = 0.1734906

Figure S6. Unit-cell parameters, the corrected observed and calculated peak positions, and the respective difference obtained from the McMaille software for the case of the Sample 1.

```
FINAL VALUES   : (STANDARD DEVIATIONS : 2nd LINE)

   ZERO     LAMBDA      A         B         C       ALPHA     BETA     GAMMA
  0.006     1.5406   5.5338    5.5261    7.8293    90.000    90.000    90.000
  0.010     0.0000   0.0008    0.0017    0.0016     0.000     0.000     0.000
RECIPROCAL CELL :    0.18071   0.18096   0.12772    90.000    90.000    90.000
VOLUME (A**3)   :       239.425

   H     K     L   TH(OBS)    TH-ZERO    TH(CALC)     DIFF.

   1     1     0    22.721     22.727     22.722      0.004
   1     1     2    32.329     32.335     32.334      0.001
   0     2     1    34.405     34.411     34.391      0.019
   1     2     1    38.125     38.131     38.145     -0.015
   2     0     2    39.866     39.872     39.866      0.006
   2     2     0    46.396     46.402     46.406     -0.004
   2     0     3    47.870     47.876     47.875      0.001
   1     1     4    52.206     52.212     52.220     -0.008
   1     3     1    53.680     53.686     53.691     -0.005
   3     1     2    57.655     57.661     57.661      0.000
   3     1     3    64.005     64.011     64.015     -0.005
   4     0     0    67.657     67.663     67.669     -0.006
   0     0     6    72.355     72.361     72.359      0.001
   4     2     0    77.005     77.011     77.026     -0.015
   4     2     2    81.562     81.568     81.542      0.025

  M(20) =      46.44
  F(20) =      29.82 (  0.0058, 115)

 Final Rp on the .prf =   0.1901640
```

Figure S7. Unit-cell parameters, the corrected observed and calculated peak positions, and the respective difference obtained from the McMaille software for the case of the Sample 2.

FINAL VALUES : (STANDARD DEVIATIONS : 2nd LINE)

	ZERO	LAMBDA	A	B	C	ALPHA	BETA	GAMMA
	-0.015	1.5406	5.5405	5.5367	7.8243	90.000	90.134	90.000
	0.008	0.0000	0.0007	0.0008	0.0015	0.000	0.009	0.000
RECIPROCAL CELL :			0.18049	0.18061	0.12781	90.000	89.866	90.000
VOLUME (A**3) :			240.021					

H	K	L	TH(OBS)	TH-ZERO	TH(CALC)	DIFF.
1	0	1	19.646	19.631	19.639	-0.008
0	0	2	22.755	22.740	22.712	0.029
1	1	1	25.448	25.433	25.429	0.004
1	0	-2	27.835	27.820	27.864	-0.044
1	1	2	32.352	32.337	32.346	-0.009
0	0	3	34.397	34.382	34.357	0.025
1	0	3	38.139	38.124	38.141	-0.017
0	2	2	39.875	39.860	39.860	0.000
1	1	-3	41.560	41.545	41.535	0.010
1	1	3	41.648	41.633	41.601	0.032
1	2	2	43.223	43.208	43.222	-0.014
0	0	4	46.389	46.374	46.382	-0.008
2	2	1	47.872	47.857	47.865	-0.008
3	0	0	49.311	49.296	49.302	-0.006
1	2	3	50.846	50.831	50.828	0.003
1	3	0	52.214	52.199	52.198	0.001
1	3	-1	53.581	53.566	53.581	-0.015
1	3	-2	57.629	57.614	57.612	0.002
3	0	-3	61.405	61.390	61.396	-0.006
3	1	-3	63.942	63.927	63.893	0.034
2	2	-4	67.594	67.579	67.581	-0.002
4	0	1	68.822	68.807	68.804	0.003
0	3	4	70.053	70.038	70.036	0.003
3	1	-4	72.284	72.269	72.258	0.011
4	2	0	76.924	76.909	76.902	0.007
4	2	-1	78.027	78.012	78.010	0.002
4	2	-2	81.380	81.365	81.368	-0.002
2	4	-2	81.459	81.444	81.434	0.010
3	1	-5	82.484	82.469	82.493	-0.024
1	3	-5	82.603	82.588	82.591	-0.003
3	1	5	82.725	82.710	82.714	-0.004
4	0	-4	85.819	85.804	85.794	0.010
0	4	4	85.964	85.949	85.960	-0.011
4	2	-3	86.918	86.903	86.923	-0.020
2	4	-3	87.033	87.018	87.003	0.015

M(20) = 19.95
F(20) = 16.73 (0.0137, 98)

Final Rp on the .prf = 0.2305329

Figure S8. Unit-cell parameters, the corrected observed and calculated peak positions, and the respective difference obtained from the McMaille software for the case of the Sample 3.

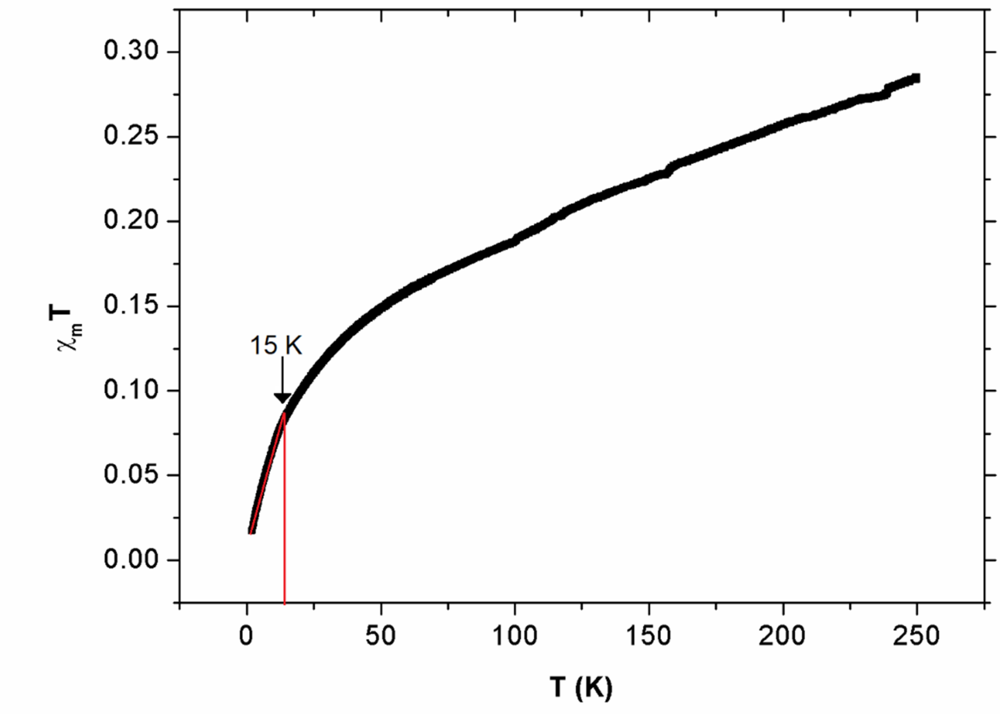

Figure S9. $\chi_m T$ vs. T curve showing the Néel temperature for the Sample 1.

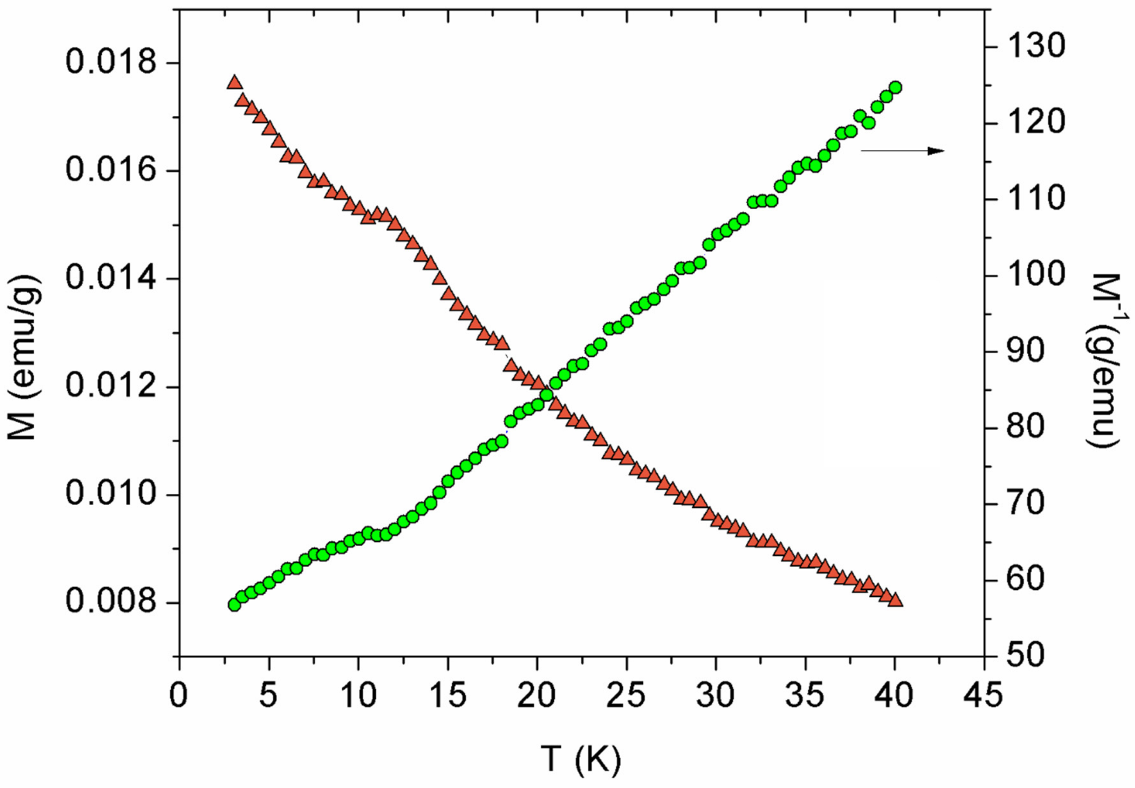

Figure S10. Variation of M along with M^{-1} vs. temperature for Sample 2 from 2 to 40 K.

References

1. ICSD, Inorganic Crystal Structure Database. Available online: https://bdec.dotlib.com.br/ (accessed on 1 December 2017).
2. Smith, G.S.; Snyder, R.L. FN: A Criterion for Rating Powder Diffraction Patterns and Evaluating the Reliability of Powder-Pattern Indexing. *J. Appl. Crystallogr.* **1979**, *12*, 60–65.
